# Factors associated with late presentation for HIV/AIDS care in Harare City, Zimbabwe, 2015

**DOI:** 10.1186/s12889-016-3044-7

**Published:** 2016-05-03

**Authors:** Howard Nyika, Owen Mugurungi, Gerald Shambira, Notion Tafara Gombe, Donewell Bangure, More Mungati, Mufuta Tshimanga

**Affiliations:** Department of Community Medicine, University of Zimbabwe, Harare, Zimbabwe; Ministry of Health and Child Care, AIDS and TB Unit, Harare, Zimbabwe

**Keywords:** HIV/AIDS, Late presentation, Harare City, Zimbabwe

## Abstract

**Background:**

Despite widespread awareness and publicity concerning Human Immunodeficiency Virus (HIV) care and advances in treatment, many patients still present late in their HIV disease. Preliminary review of the Antiretroviral Therapy (ART) registers at Wilkins and Beatrice Road Hospitals, both located in Harare, indicated that 67 and 71 % of patients enrolled into HIV/AIDS care presented late with baseline CD4 of <200 cells/uL and/or WHO stage 3 and 4 respectively. We therefore sought to explore factors associated with late presentation in Harare City.

**Methods:**

We conducted a 1:1 unmatched case control study where a case was an HIV positive individual (>18 years) with a baseline CD4 of <200/uL or who had WHO clinical stage 3 or 4 at first presentation to OI/ART centres in 2014 and; a control was HIV positive individual (>18 years) who had a baseline CD4 of >200/uL or WHO clinical stage 1 or 2 at first presentation in 2014. Written informed consent was obtained from all study participants.

**Results:**

A total of 268 participants were recruited (134 cases and 134 controls). Independent risk factors for late presentation for HIV/AIDS care were illness being reason for test (Adjusted Odds Ratio [aOR] =7.68, 95 % CI = 4.08, 14.75); Being male (aOR = 2.84, 95 % CI = 1.50, 5.40) and; experienced HIV stigma (aOR = 2.99, 95 % CI = 1.54, 5.79). Independent protective factors were receiving information on HIV (aOR = 0.37, 95 % CI = 0.18, 0.78) and earning more than US$250 per month (aOR = 0.32, 95 % CI = 0.76, 0.67). Median duration between first reported HIV positive test result and enrolment into pre-ART care was 2 days (Q_1_ = 1 day; Q_3_ = 30 days) among cases and 30 days (Q_1_ = 3 days; Q_3_ = 75 days) among controls.

**Conclusion:**

Late presentation for HIV/AIDS care in Harare City was a result of factors that relate to the patient’s sex, reason for getting a test, receiving HIV related information, experiencing stigma and monthly income. Based on this evidence we recommended targeted interventions to optimize early access to testing and enrolment into care.

## Background

Human Immunodeficiency Virus (HIV) is a lentivirus that causes acquired immunodeficiency syndrome, a condition in humans in which the immune system fails to contain life-threatening infection, which would otherwise be dealt with in non-infected individuals [1]. Sub-Saharan Africa remains the hardest hit region, with estimates ranging up to 22.5 million people living with the virus. Since 2004, there has been a rapid scale up in provision of antiretroviral therapy (ART) and close to 37 % of those in need had access to it [2]. Despite significant investment in increasing awareness concerning HIV care and advances in treatment, many patients still present late in their HIV disease with either an AIDS defining illness or a CD4 count of <200 cells/uL [3]. Clients presenting late for care have been found to have less favorable outcomes than those who initiate early [4]. HIV positive individuals with advanced HIV disease at the time of ART initiation are susceptible to treatment failure, pose a significant financial burden on the health system and have a higher likelihood of early mortality [5-8].

In addition, research suggests that a higher cumulative risk of HIV transmission by late presenters exists, thus posing a significant burden to other individuals, considering that earlier presentation and initiation on ARVs might lead to viral load suppression thereby reducing risk of transmission [5].

A cross sectional study conducted at a Ugandan rural clinic reported that approximately 40 % of the ART patients presented with WHO stage 4 infections and male sex and poor education were significant risk factors for late presentation [9]. This might result in switching to more expensive second line regimens and increased risk of transmission to other individuals [10].

### Background of the study

Zimbabwe adopted the 2013 World Health Organization antiretroviral therapy guidelines in September 2013. Previously, 2010 WHO guidelines had been in use. During the conduct of the study, the 2013 guidelines were still in use and were used as the basis for enrolling patients into care. The guidelines, state that patients be enrolled into care with CD4 < 500 cells/uL.

Harare City is the capital of the Republic of Zimbabwe and is the largest city in the country. The city is situated to the north east of Zimbabwe in Harare Metropolitan Province and has an estimated population of 1 860 219 residing in six divisional districts [11]. The city has 12 polyclinics, seven primary care clinics, 15 satellite clinics, 6 family health service clinics, 4 dental clinics and 2 infectious disease hospitals, namely Beatrice Road Hospital and Wilkins Hospital.

The City also has a specialized Genito-Urinary Centre for treatment of STIs located at Wilkins Hospital. Facilities in the city offer a wide range of programmes including TB/HIV Care, PMTCT, Reproductive Health and STI Prevention and Treatment. Programme data indicates that the leading cause of death in the city in 2014 were HIV-related illnesses (39 %) [12]. Comprehensive OI/ART services have been decentralized to 90 % of the clinics from the two infectious disease hospitals and the city recorded an increase to 86 % of all HIV positive pregnant women accessing care for Prevention of Mother to Child Transmission -PMTCT (Option B+) at the end of 2014 [12].

Late presentation to HIV/AIDS care is of importance from both a clinical and public health point of view in Harare City. In 2014, a total of a total of 797 and 1098 new adult in initiations were done at Wilkins and Beatrice Road Hospitals respectively. Preliminary review of the ART registers at both institutions indicated that 67 and 71 % of patients presented late with baseline CD4 of <200 cells/uL or WHO stage 3 and 4 despite adoption of guidelines recommending initiation at CD4 < 500 cells/uL.

## Methods

A 1:1 unmatched case control study was conducted to determine factors associated with late presentation to HIV/aids Care. This was a retrospective study design, which considered study participants on the basis of their outcome status (late or early presentation to HIV/AIDS care). Once the exposures had been ascertained and compared (by computing odds ratios), the differences between cases and controls were tested for statistical significance.

The selection of participants was done between January and March 2015, while interviews took place from 1 to 31^st^ of May 2015.

**Case:** is an HIV positive individual (≥18 years) with a baseline CD4 count of <200/uL or who had WHO clinical stage 3 or 4 at the time of first presentation to Wilkins and Beatrice Road Opportunistic Infections clinics between January and December 2014.

**Control**: is HIV positive individual (≥18 years) who had a baseline CD4 count of >200/uL or WHO clinical stage 1 or 2 at the time of first presentation to Wilkins and Beatrice Road Opportunistic Infections clinics between January and December 2014.

### Working definitions

Late presentation from an immunological point of view is enrollment into HIV/AIDS care with a CD4 count of <200 cells/uL. Enrollment into care is being entered into the Pre-ART register at a health facility and assigned an OI/ART number. Clinically**,** late presentation is defined as enrolment into care with an AIDS defining illness as prescribed by the World Health Organization and is classified as Stage 3 and 4. Clinical staging is done by the observing clinician and recorded on the patient’s Opportunistic Infections Care Booklet at presentation to the facility.

### Key informants

These were HIV program managers and facility managers, and had specific knowledge about HIV program in Harare City. They included Beatrice and Wilkins Hospitals Medical Superintendents, Hospital Matrons of the two institutions, the Sisters in Charge of the Opportunistic Infections at both hospitals and a Primary Care Counsellor at each institution.

### Patient record review

The patients OI/ART Care booklets (“the Green book”) for each participant i.e. both cases and controls were reviewed for the following

- To ascertain baseline CD4 count at first presentation to the facility after an HIV positive test.
- To ascertain the WHO clinical staging at first presentation to OI/ART clinics.

Patients on ART at Wilkins and Beatrice Road Hospitals who were available on the study days, and who agreed to participate and were well enough to take part, were included in the study.

### Sample size determination

Sample size was calculated using Fleiss formula in the StatCalc™ function of Epi Info® 7.

$$ {\mathrm{P}}_1 = {\mathrm{p}}_2\left(\mathrm{OR}\right)/1 + \left[{\mathrm{p}}_2\left(\mathrm{OR} - 1\right)\right] $$

Where:

- p_1_ = proportion of exposed with disease
- p_2_ = proportion of unexposed with disease
- OR = Calculated odds ratio form previous study [13]

A 1:1 unmatched case control study, with a 95 % Confidence Interval and 80 % power was conducted in Harare City. A minimum sample size of 122 cases and 122 controls was calculated. Assuming a 10 % non-response rate, the minimum total sample size was 268. Therefore the minimum sample size calculated was 134 cases and 134 controls.

A total of 268 records were reviewed i.e. every participant’s record. A total of 8 key informants were purposively recruited into the study.

### Sampling

The OI/ART register which captures all patients enrolled into care and had been allocated an OI/ART number was used as the sampling frame. Proportional sampling was done in accordance with the enrolled patients at each facility. Cases were randomly recruited from OI/ART register into the study using random number tables. Controls were randomly recruited from the OI/ART register using the same method after creating a separate list extracted from the same register.

Key informants were purposively selected at the two institutions due to their knowledge on HIV/AIDS programming, management and performance monitoring and evaluation. A desk review for all the 268 records (134 cases and 134 controls) was done.

### Pretesting data collection instrument

A pre-tested interviewer administered, semi-structured, questionnaire was used to collect data from cases and controls. Checklists were used to identify baseline CD4 count and baseline WHO clinical stage at first presentation. An interview guide for key informants was used to elicit information on the city’s HIV program; from inputs, processes, outputs and outcome.

The questionnaire was pretested at Parirenyatwa Central Hospital Opportunistic Infections clinic to check for appropriateness and structure of questions, and whether the intended data was being collected. The time taken to administer the questionnaire was also considered.

Questionnaires were checked for completeness and internal consistency before being created in Epi info version 7 for data analysis. The Epi Info software was used to analyse quantitative data. Means, frequencies, proportions, odds ratios (OR), and their 95 % confidence intervals (CI), were generated. Odds ratio (OR) that did not include the value 1 in the 95 % confidence interval were considered statistically significant. Forward stepwise logistic regression analysis was done to determine independent factors associated with late presentation. Qualitative data were sorted and analyzed thematically.

In terms of results utilization, written reports were given to Beatrice and Wilkins Infectious Diseases Hospital Medical Superintendents, Director City Health Department, Director AIDS and TB Unit and HSO. Presentation of results was done to the Director of Health Services Harare City and hospital superintendents.

## Results

**Fig. 1:**
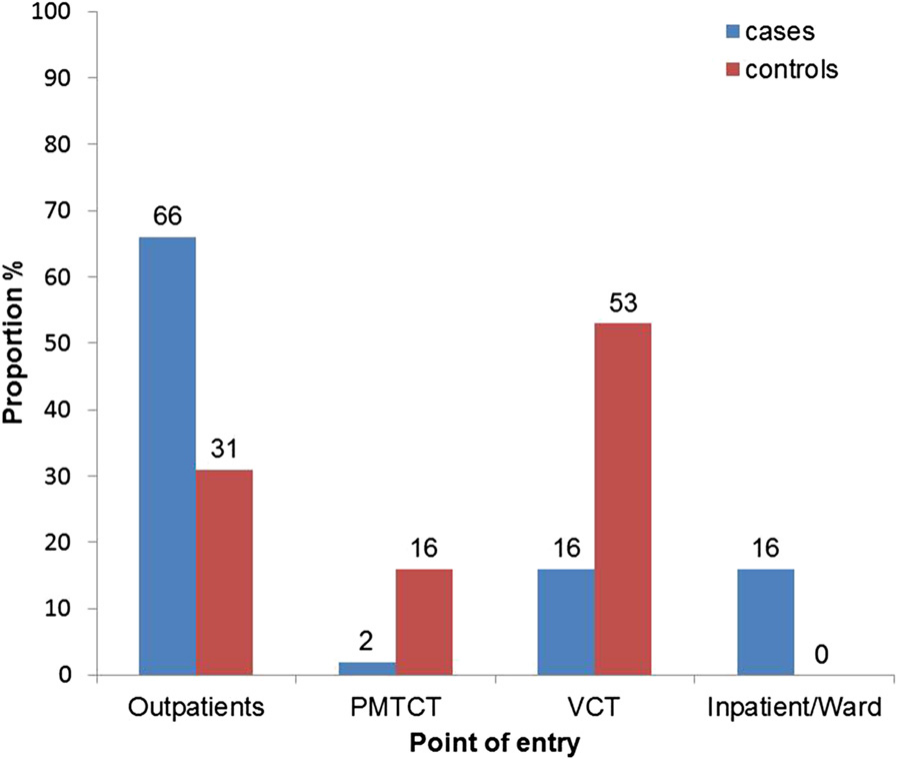
Point of entry for HIV/AIDS care, Harare City, 2015

A total of 134 cases and 134 controls were recruited into the study. Cases and controls were comparable in terms of socio-demographic characteristics except for sex and religion where cases were more likely to be male and subscribe to an apostolic sect (Table 1). The majority of cases (66 %) enrolled through the outpatient department and the least (2 %) enrolled through the PMTCT programme. The majority of controls (53 %) entered through voluntary counseling and testing (New Start Centre) offered at both institutions (Fig. 1).

**Table 1:** Socio-demographic characteristics of study participants, Harare City, Zimbabwe, 2015

Variable	Category	Cases *n* = 134 (%)	Controls *n* = 134 (%)	*p*-value
Sex	Male	77 (57)	42 (31)	<0.01
	Female	57 (43)	92 (69)	
Age	<20	2 (1)	0 (0)	0.42
	20–29	31 (23)	38 (28)	
	30–39	59 (44)	60 (45)	
	40–49	30 (22)	29 (22)	
	50+	12 (9)	7 (5)	
Median age		35 (Q_1_ = 30, Q_3_ = 40)	34 (Q_1_ = 30, Q_3_ = 43)	
Marital status	Single	24 (18)	17 (13)	0.32
	Divorced	18 (13)	18 (13)	
	Married	70 (52)	78 (58)	
	Widowed	22 (16)	21 (16)	
Place of residence	High density surbub	89 (66)	89 (66)	0.91
	Medium density	30 (22)	31 (24)	
	Low density surbub	15 (11)	14 (10)	
Highest level of education	None	3 (2)	1 (1)	0.08
	Primary	27 (20)	18 (13)	
	Secondary	86 (64)	99 (74)	
	Tertiary	18 (14)	16 (12)	
Religion	Pentecostal	42 (33)	54 (40)	<0.05
	Apostolic	47 (35)	27 (21)	
	Orthodox	20 (15)	16 (12)	
	Traditional	7 (8)	9 (8)	
	Protestant	13 (9)	25 (19)	
	None	5 (3)	3 (1)

**Fig. 2:**
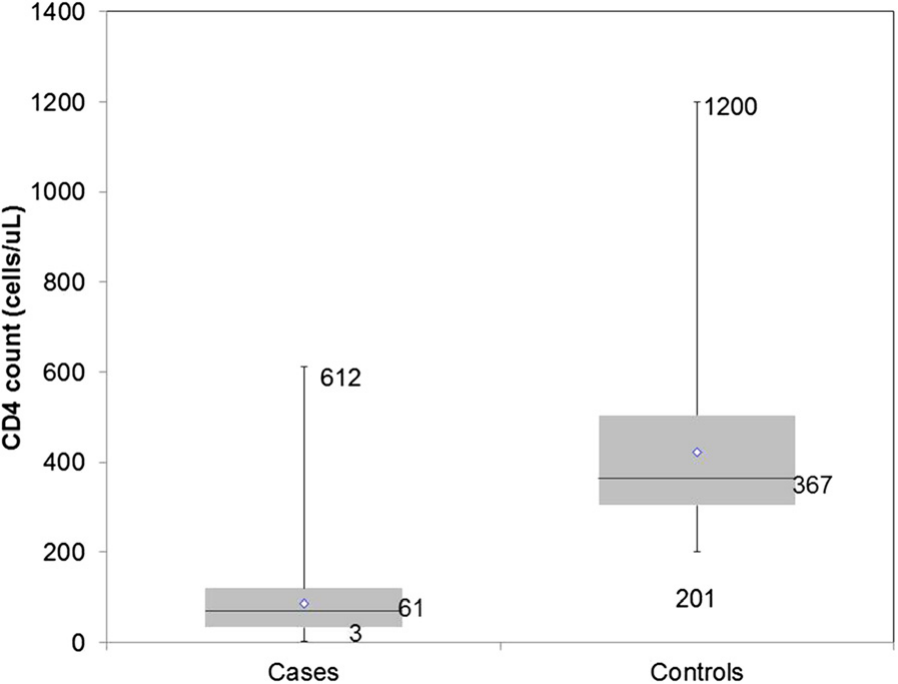
Baseline CD4 cell count at first presentation for HIV/AIDS care in Harare City, 2015 (*n* = 268)

**Fig. 3:**
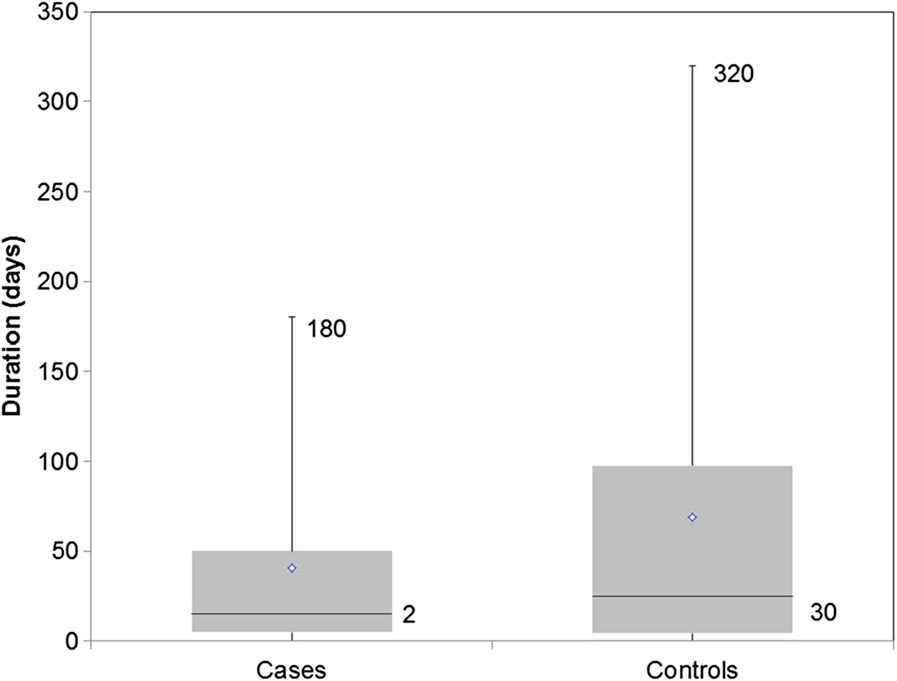
Duration between reported first HIV positive test and enrollment into care, Harare City, 2015 (*n* = 268)

The median baseline CD4 count at first presentation to the OI/ART clinic among cases was 61 cells/uL (Q_1_ = 34; Q_3_ = 110), with the maximum CD4 count value being 612 cells/uL of blood and the minimum value being 3 cells/uL of blood. Among the controls, the median CD4 count was 367 cells/uL (Q_1_ = 301; Q_3_ = 505), the maximum value being 1200 cell/uL and the least value was 201 cells/uL (Fig. 2). The median duration between testing positive (reported) and enrolling into care among cases was 2 days (Q_1_ = 1; Q_3_ = 30) with a maximum delay of 180 days. The median duration from testing positive and enrolling into care among controls was 30 days (Q_1_ = 3; Q_3_ = 75) and the maximum duration was 320 days (Fig. 3).

Forward stepwise logistic regression was conducted to determine independent factors associated with late presentation for HIV/AIDS Care in Harare City. Independent risk factors for late presentation for HIV/AIDS care were illness being reason for test (aOR = 7.68, 95 % CI = 4.08, 14.75); Being male (aOR = 2.84, 95 % CI = 1.50, 5.40) and; experienced HIV stigma (aOR = 2.99, 95 % CI = 1.54, 5.79). Independent protective factors were receiving information on HIV (aOR = 0.37, 95 % CI = 0.18, 0.78), earning more than US$250 per month (aOR = 0.32, 95 % CI = 0.76, 0.67) (Table 2).

**Table 2:** Independent factors for late presentation for HIV/AIDS care, Harare City, Zimbabwe, 2015

Variable	aOR	95 % C.I	*P*-value
Illness reason for HIV test	7.68	4.08, 14.75	<0.01
Experienced HIV stigma	2.99	1.54, 5.79	<0.01
Being male	2.84	1.50, 5.40	<0.01
Received information on HIV	0.37	0.18, 0.78	0.01
Earned more than $250 per month	0.32	0.76, 0.67	<0.01

The HIV focal person mentioned that all health facilities were implementing the 2013 WHO OI/ART Guidelines, which were fully adopted in January 2014. Hospital matrons indicated that 90 % of all staff which include doctors, nurses and counsellors had all been trained in HIV Intergrated Training focusing on support and supervision and monitoring and evaluation. The city implements mentorship visits on a "week in week out" basis. The average waiting time for patients was less than 10 min per facility.

A total of 27 sites initiate ART and have CD4 machines onsite. Support and supervision is conducted once every quarter to all facilities. Majority of the key informants (8) mentioned that fear of HIV related stigma was the main reason for late presentation for care. The least number of respondents (4) mentioned that lack of disclosure to relatives could be a possible reason for late presentation for care.

## Discussion

CD4 count determination is important when making decisions on starting ART using the 2013 guidelines. In this study, the presentation of cases within median CD4 count of 61 cells/uL reaffirms the fact that CD4 count at presentation by patients in resource limited settings remains critically low and is an indication of late presentation [14]. Furthermore, the median delay for care after testing positive varied between cases and controls (2 days and 30 days respectively). This is consistent with what was found out in Kenya during a community testing campaign where there was a delay between testing and linkage to care [7]. The delay was attributed to lack of training on the part of service providers. The period between testing and linking to care is critical as it may result in loss to follow up of patients, therefore jeopardizing their health.

Having an HIV test due to illness was an independent risk factor associated with late presentation to HIV/AIDS care in Harare City. This factor was the most significant in this study. This finding is similar to what Abaynew et al., found out where illness at first HIV positive test was significantly associated with late presentation (OR = 2.61, 95 % CI = 1.26, 5.43) [13]. This is also supported by evidence from India where 83 % of participants classified as late presenters had an AIDS defining illness or a sexually transmitted infection [14]. This could mean that individuals are only coming to testing centers primarily when they have developed AIDS related conditions. The public health message derived from this finding is that late presentation might be a direct consequence of late diagnosis of HIV and ultimately late linkage to care and treatment.

Experiencing stigma as a result of being HIV positive was independently associated with presenting late to HIV/AIDS care (OR = 2.99, 95 % CI = 1.54, 5.79). This may be due to the fact there might be loss of material and/or emotional benefits if one’s status is known in the community, particularly in Harare City, where some occupations such as pirate taxi driving and vending are accompanied by verbal abuse thus patients who are in these occupations are stigmatized by their peers in the same trades [15]. This finding gives credence to overwhelming evidence that HIV related stigma is a hindrance to early presentation for care in Africa and beyond [16–18].

In this study, being male was an independent risk factor for presenting late for HIV/AIDS care in Harare City. Several studies have also reported low uptake of HIV services among men as compared to women in different settings [19–22]. This finding supports the notion that men generally do not seek testing and counseling services on a routine basis, thus leading to late diagnosis of HIV when they are already on late stage disease progression. Haskew et al., reported that men had 1.4 times higher odds of presenting to the clinic late in the course of HIV infection compared to women [9]. This finding may also be explained in the general sense, that women have more contact with the health facility on a more routine basis than men. Women attended antenatal care clinics and other maternal and child health related clinics hence are more likely to be tested as service provided in the continuum of care. The introduction of Option B+ on Harare City has also increased the likelihood of early presentation among women. Mujumdar et al., in a study of an HIV-1 infected population in rural India found out that males were twice as likely to present late more than females [14].

Patients are likely to access information about HIV through the visual and print media and crucially when they go for routine HIV testing and counseling at VCT centres situated around the city or at outpatient departments at clinics. Ndawitz et al., in Cameroon reported that living in a region with higher comprehensive knowledge of HIV/AIDS was associated with not initiating ART late (aOR = 0.8, 95 % CI = 0.6, 1.0) [21]. This suggests that information dissemination hugely increases awareness of the risks associated with HIV and this provides an opportunity for public health practitioners to craft messages for specific at risk populations in Harare City. The print and electronic media also play an active role in reaching out to communities and facilitate behavior change.

In this study, those who earned more than US$250 were less likely to present late for HIV/AIDS care. It is plausible that when income is high, this might also reciprocate into better access to health services. Louis et al., in Haiti reported that poverty i.e. earning below the poverty datum line was significantly associated with late presentation for HIV care at ART initiating clinics [22]. Furthermore, those who earn below the poverty datum line are likely to be self-employed hence do not have access to work related testing and counseling services, which are enjoyed by their formally employed counterparts. Also, those who earn less are likely to be more preoccupied with selling their wares that they may not be cognizant of the need to visit the health facility regularly [23]. In the study by Louis, it was also reported that harsh poverty was a theme of all of the respondents. With this in mind, programme managers in Harare City ought to find strategies to encourage routine testing and counseling.

### Limitations of the study

The study relied on patients’ self-reporting of historical events, thus creating recall bias. This was minimized by selecting patients initiated in the 2014 cohort, who were more likely to remember events more vividly. Characteristics of patients who never attended OI/ART clinics at Beatrice and Wilkins Hospital could not be established, which could have affected generalizability of the study results. This was minimized by selecting a large sample size and proportionately recruiting respondents from the study sites.

## Conclusions

Multiple factors were associated with late presentation to HIV/ADIS care in Harare City. Getting an HIV test due to illness, being male and experiencing HIV related stigma were identified independent risk factors associated with late presentation in the city. Receiving information on HIV and earning more than US$250 monthly were independent protective factors associated with late presentation for HIV/AIDS care in the city. The findings of this study guided AIDS and TB programme managers, particularly the Prevention, Testing and Counseling section on addressing late presentation in Harare City.

## Ethical consideration

Ethical clearance was obtained from the Joint Research Ethics Committee for College of Health Sciences and Parirenyatwa Group of Hospitals (JREC 131/15). Permission to conduct the study was obtained from Harare City Council Health Department, Health Studies Office and the Medical Research Council of Zimbabwe (MRCZ B/875). Written informed consent was obtained from study participants. The completed questionnaires were secured in a locker. Study participants were treated with dignity, regardless of race, gender, political or religious affiliation. No names or addresses of participants were used in the study. Confidentiality was maintained throughout the study. Participation was voluntary and there were no financial gains for participating in the study.

## Consent to publish

Consent to publish was obtain from the Medical Research Council of Zimbabwe and also obtained from study participants.

## Availability of data and materials

Datasets and materials available via Dropbox® and can be shared upon request.
